# Expression of voltage-gated sodium channel Nav1.5 in non-metastatic colon cancer and its associations with estrogen receptor (ER)-β expression and clinical outcomes

**DOI:** 10.1186/s40880-017-0253-0

**Published:** 2017-11-09

**Authors:** Jianhong Peng, Qingjian Ou, Xiaojun Wu, Rongxin Zhang, Qian Zhao, Wu Jiang, Zhenhai Lu, Desen Wan, Zhizhong Pan, Yujing Fang

**Affiliations:** 1 Department of Colorectal Surgery, State Key Laboratory of Oncology in South China, Collaborative Innovation Center for Cancer Medicine, Sun Yat-sen University Cancer Center, 651 Dongfeng Road East, Guangzhou, 510060 Guangdong P. R. China; 2 Department of Experimental Research, Sun Yat-sen University Cancer Center, Guangzhou, 510060 Guangdong P. R. China; 3 Department of Statistics, School of Public Health, Guangzhou Medical University, Guangzhou, 510182 Guangdong P. R. China

**Keywords:** Nav1.5, Estrogen receptor-β, Colon cancer, Clinical outcome

## Abstract

**Background:**

Voltage-gated sodium channel 1.5 (Nav1.5) potentially promotes the migratory and invasive behaviors of colon cancer cells. Hitherto, the prognostic significance of Nav1.5 expression remains undetermined. The present study aimed to explore the associations of Nav1.5 expression with clinical outcomes and estrogen receptor-β (ER-β) expression in non-metastatic colon cancer patients receiving radical resection.

**Methods:**

A total of 269 consecutive patients with pathologically confirmed stages I–III colon cancer who underwent radical resection were selected. Nav1.5 and ER-β expression was detected by using immunohistochemistry (IHC) on tissue microarray constructed from paraffin-embedded specimens. IHC score was determined according to the percentage and intensity of positively stained cells. Statistical analysis was performed with the X-tile method, *k* coefficient, Chi square test or Fisher’s exact test, logistic regression, log-rank test, and Cox proportional hazards models.

**Results:**

We found that Nav1.5 was commonly expressed in tumor tissues with higher mean IHC score as compared with matched tumor-adjacent normal tissues (5.1 ± 3.5 vs. 3.5 ± 2.7, *P* < 0.001). The high expression of Nav1.5 in colon cancer tissues was associated with high preoperative carcinoembryonic antigen level [odds ratio (OR) = 2.980; 95% confidential interval (CI) 1.163–7.632; *P* = 0.023] and high ER-β expression (OR = 2.808; 95% CI 1.243–6.343; *P* = 0.013). Log-rank test results showed that high Nav1.5 expression contributed to a low 5-year disease-free survival (DFS) rate in colon cancer patients (77.2% vs. 92.1%, *P* = 0.048), especially in patients with high ER-β expression tumor (76.2% vs. 91.3%, *P* = 0.032). Analysis with Cox proportional hazards model demonstrated that high Nav1.5 expression [hazard ratio (HR) = 2.738; 95% CI 1.100–6.819; *P* = 0.030] and lymph node metastasis (HR = 2.633; 95% CI 1.632–4.248; *P* < 0.001) were prognostic factors for unfavorable DFS in colon cancer patients.

**Conclusions:**

High expression of Nav1.5 was associated with high expression of ER-β and indicated unfavorable oncologic prognosis in patients with non-metastatic colon cancer.

## Introduction

Colorectal cancer is the fifth leading cause of cancer-related death in China, with a total of 191,000 deaths projected for 2015 [1]. Depending on the stage at diagnosis, there are several new treatment patterns for colon cancer recently, including robotic surgery and maintenance treatment for selected patients [2, 3]. For non-metastatic colon cancer, the most effective treatment is surgery, with adjuvant chemotherapy or radiation therapy as required [4]. However, 20%–30% of these patients would ultimately develop metastatic disease despite receiving radical treatment [5, 6]. Although risk factors including T4 tumor, elevation of preoperative carcinoembryonic antigen (CEA) level, and presence of lymphovascular or perineural invasion and mesenteric tumor nodules have been found to partially account for the poor clinical outcome, it still seems insufficient to fulfill the clinical requirements of precision medicine [7, 8]. Therefore, exploring new biomarkers is imperative to distinguish subgroups with recurrence risks and individualize the therapy regimens.

Recently, the mounting evidence has demonstrated that the abnormal expression of some ion channels in cancer cells, as compared with those in the corresponding non-cancer cells, associated with cell proliferation, resistance to apoptosis, cell motility, and extracellular matrix invasion [9, 10]. Voltage-gated sodium channels (VGSCs), as transmembrane glycoproteins, mainly mediate the rapid upstroke of the action potential in excitable tissues such as the heart, skeletal muscle, and brain. Their dysfunction was initially identified to contribute to cardiac conduction disease [11, 12]. With respect to the non-excitable tissues, VGSCs could also be detected in cancer cells, where they might increase cancer malignancy, including promoting metastasis development [13–15]. Especially, voltage-gated sodium channel 1.5 (Nav1.5) encoded by sodium voltage-gated channel alpha subunit 5 (*SCN5A*) gene has been found to be overexpressed in highly invasive breast cancer cell line [16, 17]. Increasing evidence indicated that Nav1.5 was the key regulator to the oncogenic behavior of colon cancer cells [18, 19]. Mechanistic studies further revealed that Nav1.5 mainly enhanced cancer cell invasiveness by functionally interacting with Na^+^/H^+^ exchanger type 1 (NHE-1) to degrade the extracellular matrix and increasing Src kinase activity to promote cell invadopodia [20, 21]. In addition, the expression of Nav1.5 was also regulated by hormones and growth factors, such as β-estradiol (E2) and vascular endothelial growth factor (VEGF) [22]. Our previous studies had shown that estrogen receptor-β (ER-β) was the dominant receptor in human colonic mucosa and commonly expressed in colon cancer tissues [23, 24]. Thereby, we hypothesized that the expression of ER-β might be associated with the expression level of Nav1.5 in colon cancer. Moreover, the association of Nav1.5 expression with clinical outcomes and ER-β expression in colon cancer have not been fully elucidated in previous studies.

Thus, our present study aimed to explore prognostic predicting value of Nav1.5 expression and relationship of Nav1.5 and ER-β expression based on the long-term survival outcome of the patients with non-metastatic colon cancer.

## Patients and methods

### Patient selection

Medical records of consecutive patients from the Sun Yat-sen University Cancer Center (Guangzhou, China) between June 1997 and April 2003 were retrospectively investigated. All included patients met the following inclusion criteria: (1) histologically confirmed colon adenocarcinoma; (2) pathologic stage I–III diseases according to the 7th edition of Union for International Cancer Control (UICC) tumor-node-metastasis (TNM) classification; and (3) radical resection for colon tumor. The exclusion criteria were as follows: (1) neoadjuvant therapy before surgery; (2) confirmed metastasis preoperatively; (3) the existence of multiple primary colorectal cancers; or (4) other active malignancy (except for basal cell carcinoma of the skin). Patient demographic and clinicopathologic characteristics were retrieved from the medical records, and follow-up data were collected from the tracking system. The present study was undertaken in accordance with the ethical standards of the World Medical Association Declaration of Helsinki. The study and consent procedure were approved by the Institutional Research Ethics Committee of Sun Yat-sen University Cancer Center (Approval Number: GZR-2016-071), and informed consents for using tissue samples were obtained from the patients before the initial treatment. The authenticity of this article has been validated by uploading the key raw data onto the Research Data Deposit public platform (http://www.researchdata.org.cn), with the approval number as RDDB2017000048.

### Tissue microarrays and immunohistochemistry

The tissue microarrays (TMAs) were constructed using a personal tissue array (Beecher Instruments, Sun Prairie, WI, USA). Briefly, each tissue core with a diameter of 0.6 mm was punched in the marked areas of formalin-fixed, paraffin-embedded specimens from 269 tumors and 78 matched tumor-adjacent normal tissues (surgical margin). The organized TMA blocks were sectioned into 4-μm slices that were mounted onto glass slides. After dewaxing, the slides were treated with 0.3% hydrogen peroxide and then incubated with a Nav1.5 primary antibody (1:800 dilution, rabbit polyclonal, ab56240; Abcam, Cambridge, UK) and ER-β antibody (1:500 dilution, polyclonal rabbit, ab5786; Abcam, Cambridge, UK), in a moist chamber at 4 °C overnight. Subsequently, the slides were washed with 1× phosphate-buffered saline (PBS) and treated with a biotinylated anti-rabbit secondary antibody (Zhongshan Golden Bridge Biotechnology, Beijing, China) at 37.5 °C for 30 min. The immunohistochemical (IHC) staining was completed by incubation with 3,3′-diaminobenzidine tetrahydrochloride (DAB; Dako, Glostrup, Denmark) to stain the slides.

### IHC scoring

The IHC score was determined by the semi-quantitative method according to the percentage and intensity of positively stained cells. The percentage of positively stained cells was scored as follows: 0, less than 5% positively stained cells; (1) 5%–24%; (2) 25%–49%; (3) 50%–74%; and (4) 75%–100%. The intensity was scored according to the following criteria: 0, negative staining; (1) weak staining; (2) moderate staining; and (3) strong staining. The final IHC score was generated by multiplying the percentage score with the staining intensity score. Two trained pathologists blindly evaluated all the specimens. X-tile software version 3.6.1 (Yale University School of Medicine, New Haven, CT, USA) was applied to generate the optimal cut-off value of Nav1.5 expression with respect to DFS and OS, as described previously [25]. High Nav1.5 expression grade was defined when the IHC score was greater than the optimal cut-off value and high ER-β expression grade was defined as strong staining with at least 50% positively stained cells.

### Follow-up

All patients were monitored through subsequent visits every 3 months for the first 2 years and thereafter semiannually until 5 years after radical resection. The final follow-up visit occurred in June 2016. Clinical examination, CEA test, abdominal ultrasonography, chest radiography, colonoscopy, and computed tomography (CT) scan of the chest/abdominal/pelvic were conducted to identify tumor recurrence. Overall survival (OS) was defined as the duration from radical resection to death from any cause or the last follow-up date, whereas the disease-free survival (DFS) was defined as the duration from tumor resection to disease recurrence or the last follow-up date. Patients without any event (recurrence or death) at the last follow-up date were regarded as random censoring. The reasons of lost follow-up during the study period have been carefully examined individually and assumed as random censoring as well.

### Statistical analysis

Observed concordance between the two pathologists was calculated as a percentage of tumor tissues with concordant expression grade (high and low expression) according to the cut-off IHC score. *k* coefficient was employed to measure the evaluation quality of IHC score. The clinicopathologic data were analyzed using Statistical Package for the Social Sciences (SPSS, version 17.0, Chicago, IL, USA). Student’s *t* test was used to analyze the difference in Nav1.5 expression between colon cancer and tumor-adjacent normal tissues. The relationship between Nav1.5 expression and patient characteristics was analyzed by Chi square test or Fisher’s exact test and further verified by multivariate logistic regression analysis. The OS and DFS rates were estimated by the Kaplan–Meier method and differences between the two groups were subsequently assessed by the log-rank test. Cox proportional hazards models were used to identify the prognostic factors for DFS and calculate the hazard ratios (HRs) and their confidence intervals (CIs). All statistical tests were two-sided, and the significance level was set at 0.05.

## Results

### Patient characteristics

A total of 269 patients were included in the present study. As shown in Table 1, the median age of total patients was 60 years (range 22–85 years), with 53.9% (145/269) males and 46.1% (124/269) females. With regard to the TNM stage, 61 (22.7%) patients were diagnosed with stage I colon cancer, 134 (49.8%) with stage II colon cancer, and 74 (27.5%) with stage III colon cancer. The median number of metastatic lymph nodes in stage III patients was 2 (range 1–14).

**Table 1:** Associations of clinicopathologic characteristics with Nav1.5 expression in 269 patients with colon cancer

Characteristics	Total patients	Nav1.5 low expression	Nav1.5 high expression	*P* value
Clinical parameter				
Gender				0.384
Male	145 (53.9)	18 (12.4)	127 (87.6)	
Female	124 (46.1)	20 (16.1)	104 (83.9)	
Age (years)				
≤ 60	136 (50.6)	21 (15.4)	115 (84.6)	0.532
> 60	133 (49.4)	17 (12.8)	116 (87.2)	
Tumor localization				0.503
Right-sided colon	98 (36.4)	12 (12.2)	86 (87.8)	
Left-sided colon	171 (63.6)	26 (15.2)	145 (84.8)	
Preoperative serum CEA (ng/mL)^a^				0.011
≤ 5	154 (62.6)	29 (18.8)	125 (81.2)	
> 5	92 (37.4)	6 (6.5)	86 (93.5)	
Preoperative serum CA199 (U/mL)^b^				0.947
≤ 35	180 (81.1)	25 (13.9)	155 (86.1)	
> 35	42 (18.9)	6 (14.3)	36 (85.7)	
Adjuvant chemotherapy				0.556
Yes	228 (84.8)	31 (81.6)	197 (85.3)	
No	41 (15.2)	7 (18.4)	34 (14.7)	
Pathologic feature				
Tumor size (cm)				0.863
≤ 5	138 (51.3)	19 (13.8)	119 (86.2)	
> 5	131 (48.7)	19 (14.5)	112 (85.5)	
Tumor differentiation				0.983
Well and moderate	248 (92.2)	35 (14.1)	213 (85.9)	
Poor	21 (7.8)	3 (14.3)	18 (85.7)	
T category				0.385
T1	17 (6.3)	8 (47.1)	9 (52.9)	
T2	50 (18.6)	4 (8.0)	46 (92.0)	
T3	108 (40.1)	9 (8.3)	99 (91.7)	
T4	94 (34.9)	17 (18.1)	77 (81.9)	
N category				0.545
N0	195 (72.5)	26 (13.3)	169 (86.7)	
N1-2	74 (27.5)	12 (16.2)	62 (83.8)	
TNM stage				0.836
I	61 (22.7)	11 (18.0)	50 (82.0)	
II	134 (49.8)	15 (11.2)	119 (88.8)	
III	74 (27.5)	12 (16.2)	62 (83.8)	
ER-β status^c^				0.024
Low expression	51 (19.8)	12 (23.5)	39 (76.5)	
High expression	206 (80.2)	23 (11.2)	183 (88.8)	

All data are presented as number of patients followed by percentage in the parentheses

*ER-β* estrogen receptor-β, *TNM* tumor-node-metastasis, *CEA* carcinoembryonic antigen, *CA199* cancer antigen (CA) 199

^a^The data of preoperative CEA level were available in 246 patients

^b^The data of preoperative CA199 level were available in 222 patients

^c^ER-β status was evaluated in 257 patients

### Concordance assessment of Nav1.5 and ER-β expression in colon cancer tissues

The concordance assessment results of Nav1.5 expression were available in 242 tumor tissues. Overall concordance for Nav1.5 expression grade of tumor tissues according to either pathologist was 91.3%, and the *k* coefficient was 0.788 (95% CI 0.694–0.871). The concordance assessment results of ER-β expression were available in 173 tumor tissues. Overall concordance for ER-β expression grade of tumor tissues according to either pathologist was 90.8%, and the *k* coefficient was 0.677 (95% CI 0.517–0.831). Scoring evaluation of Nav1.5 and ER-β expression in the present study was considered fair to good concordance.

### Nav1.5 expression in colon cancer and tumor-adjacent normal tissues

**Fig. 1:**
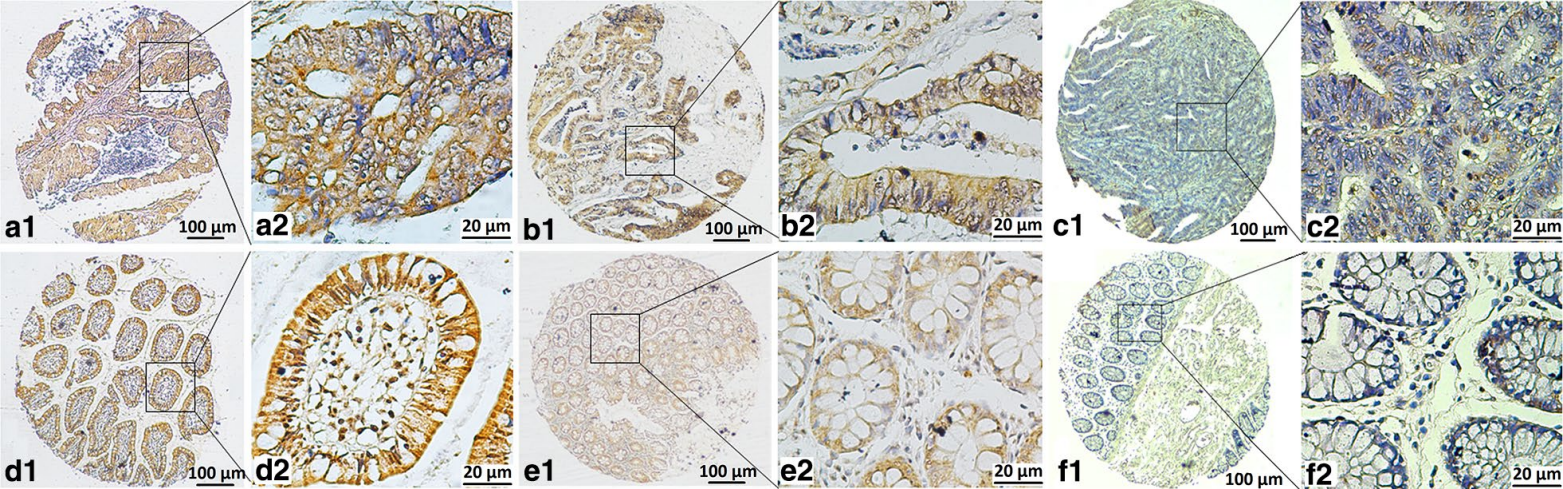
Representative immunohistochemical (IHC) staining of voltage-gated sodium channel 1.5 (Nav1.5) protein in tissue microarray (TMA). **a1**, **a2** colon adenocarcinoma with high Nav1.5 expression, with strong intensity and 100% malignant cells staining in the plasma membrane; **b1**, **b2** colon adenocarcinoma with moderate Nav1.5 expression, with moderate intensity and 75% malignant cells staining in the cell membrane; **c1**, **c2** colon adenocarcinoma with low Nav1.5 expression, with week intensity and 45% malignant cells staining in the cell membrane; **d1**, **d2** high Nav1.5 expression in tumor-adjacent normal tissue, with strong intensity and 100% epithelial cells staining in the plasma membrane; **e1**, **e2** moderate Nav1.5 expression in tumor-adjacent normal tissue, with moderate intensity and 80% epithelial cells staining in the cell membrane; **f1**, **f2** negative Nav1.5 expression in tumor-adjacent normal tissue

**Fig. 2:**
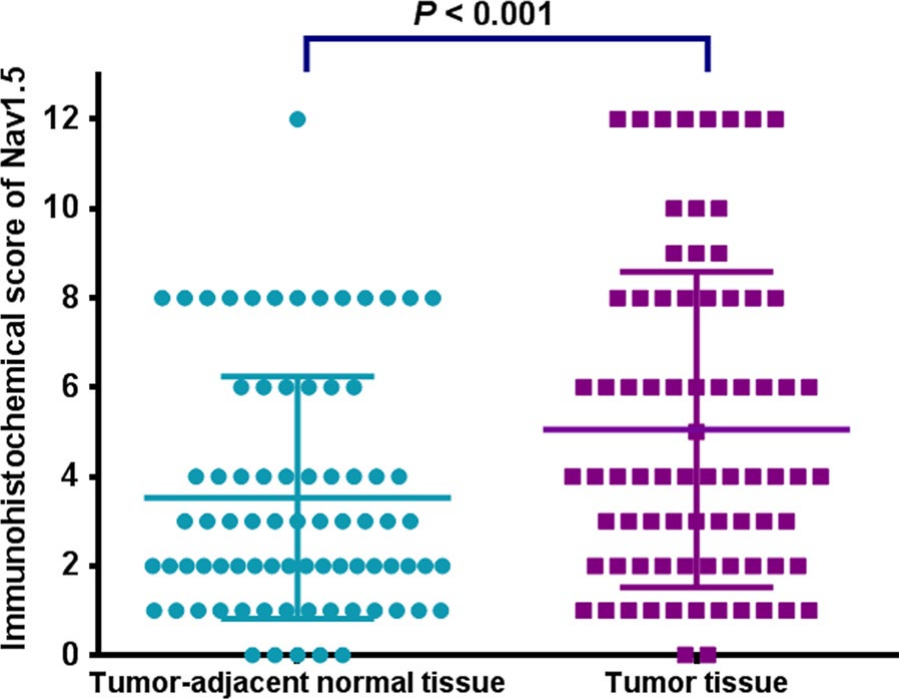
Nav1.5 expression level in colon cancer and matched tumor-adjacent normal tissues from 78 patients detected by IHC staining of TMA (5.1 ± 3.5 vs. 3.5 ± 2.7, *P* < 0.001)

As shown in Fig. 1, positive staining of Nav1.5 was mainly located in the cytoplasm of the cells, which was observed in 97.8% (263/269) of colon cancer tissues. Among 269 patients, the mean IHC score of Nav1.5 expression was significantly higher in tumor tissues than in tumor-adjacent normal tissues (5.1 ± 3.5 vs. 3.5 ± 2.7, *P* < 0.001; Fig. 2). According to the results from X-tile software, the optimal cut-off value of Nav1.5 expression was 2 at the maximum log-rank Chi square value of DFS. High expression grade (IHC score > 2) was detected in 85.9% (231/269) of colon cancer tissues but only in 52.6% (41/78) of tumor-adjacent normal tissues.

### Association of Nav1.5 expression with clinicopathologic characteristics and ER-β expression

**Fig. 3:**
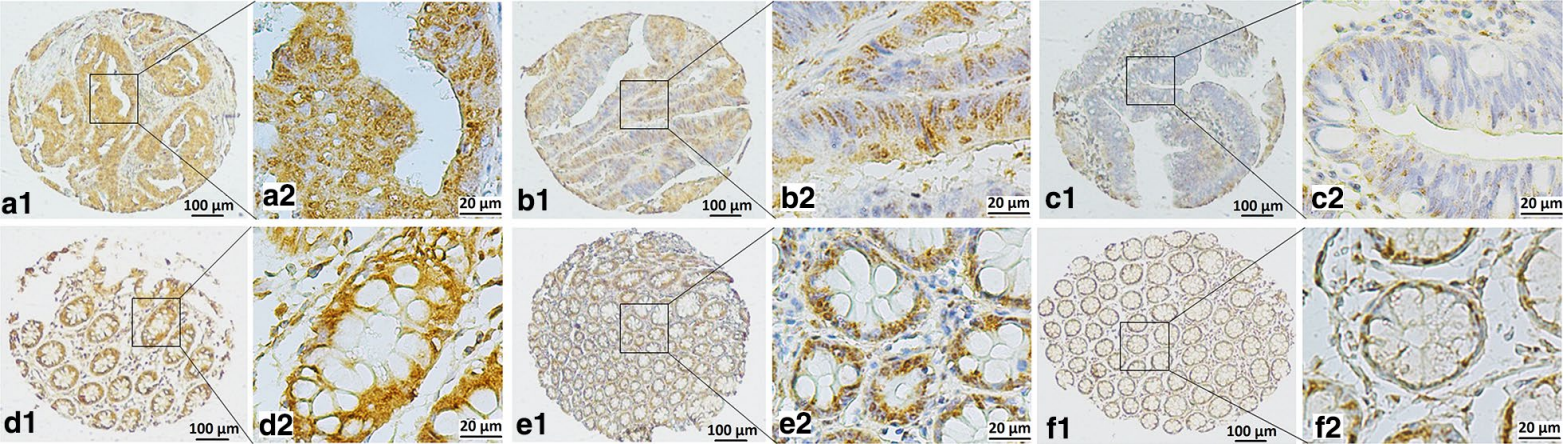
Representative TMA-IHC figures of estrogen receptor-β (ER-β) expression. **a1**, **a2** high ER-β expression in colon adenocarcinoma with strong intensity and 80% malignant cells staining in nucleus; **b1**, **b2** moderate ER-β expression in colon adenocarcinoma with strong intensity and 45% malignant cells staining in nucleus; **c1**, **c2** low ER-β expression in colon adenocarcinoma with moderate intensity and 30% malignant cells staining in nucleus; **d1**, **d2** high ER-β expression in tumor-adjacent normal tissue with strong intensity and 95% epithelial cells staining in nucleus; **e1**, **e2** moderate ER-β expression in tumor-adjacent normal tissue with strong intensity and 60% epithelial cells staining in nucleus; **f1**, **f2** low ER-β expression in tumor-adjacent normal tissue with strong intensity and 30% epithelial cells staining in nucleus

We assessed the association of Nav1.5 expression in tumor tissues with the following clinicopathologic variables: gender, age, tumor location, tumor size, tumor differentiation, preoperative CEA and CA199 levels, pathologic TNM stage, acceptance of adjuvant chemotherapy, and ER-β expression. The cut-off value of IHC score in ER-β expression was 8. As shown in Table 1, high Nav1.5 expression was associated with high preoperative serum CEA level (CEA > 5 ng/mL, *P* = 0.011). As presented in Fig. 3, the expression of ER-β was observed in the nucleus. High expression of ER-β were detected in 206 (80.2%) tumor tissues and 17 (6.6%) tumor-adjacent normal tissues, which was associated with high expression of Nav1.5 (*P* = 0.024, Table 1). In logistic regression analysis, the CEA level [multivariate odds ratio (OR) = 2.980; 95% CI 1.163–7.632; *P* = 0.023] and ER-β expression (OR = 2.808; 95% CI 1.243–6.343; *P* = 0.013) remained significantly associated with Nav1.5 expression.

### Long-term survival outcome

**Fig. 4:**
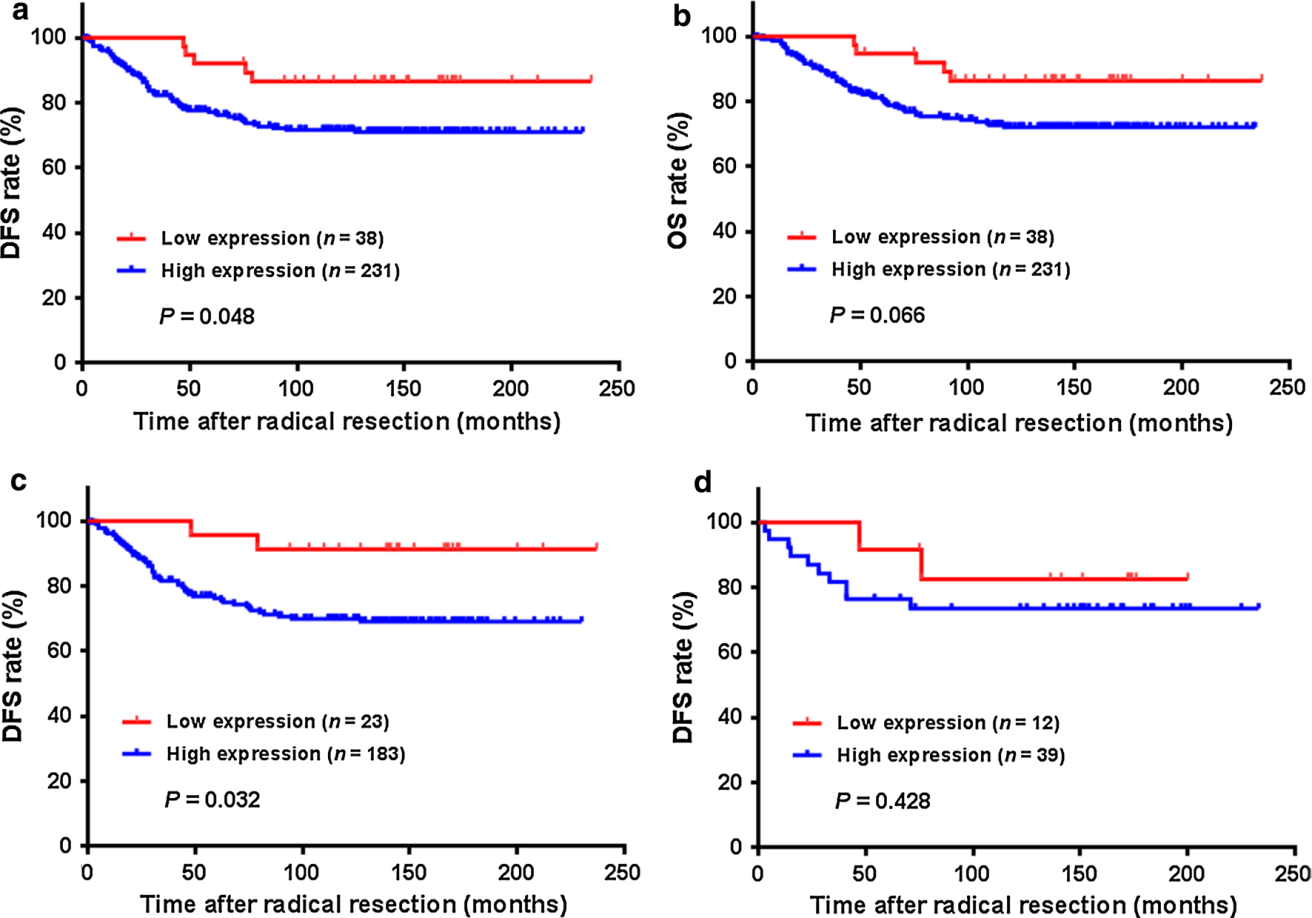
Kaplan–Meier curves comparing 5-year survival outcome of colon cancer patients with high and low Nav1.5 expression. **a** There is no significant difference in overall survival. **b** Disease-free survival (DFS) is significantly shorter in patients with high Nav1.5 expression than in those with low expression. **c** In patients with high ER-β expression colon tumor, DFS is significantly shorter in patients with high Nav1.5 expression than in those with low expression. **d** In patients with low ER-β expression colon tumor, there is no significant difference in DFS between the two cohorts

During a median follow-up of 134 months (range 1–237 months), 68 (25.3%) patients ultimately developed postoperative recurrences, and the recurrent locations were finally confirmed in 37 patients. Among them, 11 (29.8%) patients had postoperative recurrences in multiple organs, whereas 26 (70.2%) developed single organ recurrence, including 8 (30.8%) in the liver, 6 (23.1%) in the lung, 4 (15.4%) in the peritoneum, 4 (15.4%) in the bone, 2 (7.7%) in the brain, 1 (3.8%) in the ovary, and 1 (3.8%) in the abdominal wall. The 5-year DFS and OS rates were 79.3% and 83.1%, respectively, in all 269 patients. Patients with high Nav1.5 expression in tumors had significantly lower 5-year DFS rate than those with low Nav1.5 expression (77.2% vs. 92.1%, *P* = 0.048; Fig. 4a). No statistically significant difference was observed in terms of 5-year OS rate between the two groups (81.2% vs. 94.7%, *P* = 0.066; Fig. 4b). Further analysis of patients stratified by ER-β expression demonstrated that, in high ER-β expression subgroup, the patients with high Nav1.5 expression had significantly higher 5-year DFS rate than those with low Nav1.5 expression (76.2% vs. 91.3%, *P* = 0.032; Fig. 4c). However, in low ER-β expression subgroup, no statistically significant difference of 5-year DFS rates was observed between the patients with high and low Nav1.5 expression (84.2% vs. 91.7%, *P* = 0.428; Fig. 4d).

As shown in Table 2, univariate analysis revealed that high Nav1.5 expression (HR = 2.504; 95% CI 1.007–6.227; *P* = 0.048), lymph node metastasis (HR = 2.524; 95% CI 1.566–4.070; *P* < 0.001), and preoperative CEA > 5 ng/mL (HR = 1.726; 95% CI 1.049–2.841; *P* = 0.032) were associated with low 5-year DFS rate. The multivariate analysis subsequently demonstrated that high Nav1.5 expression (HR = 2.738; 95% CI 1.100–6.819; *P* = 0.030) and lymph node metastasis (HR = 2.633; 95% CI 1.632–4.248; *P* < 0.001) were prognostic factors for low 5-year DFS rate. In addition, Nav1.5 expression (HR = 4.653; 95% CI 1.131–19.142; *P* = 0.033) and lymph node metastasis (HR = 2.232; 95% CI 1.309–3.808; *P* = 0.003) remained the prognostic factors for 5-year DFS in patients with high ER-β expression colon cancer (Table 3).

**Table 2:** Univariate and multivariate Cox regression analyses of prognostic predictors for 5-year disease-free survival in 269 patients with non-metastatic colon cancer undergoing radical resection

Variables	Univariate analysis		Multivariate analysis	
	HR (95% CI)	*P* value	HR (95% CI)	*P* value
Nav1.5 expression (high vs. low)	2.504 (1.007–6.227)	0.048	2.738 (1.100–6.819)	0.030
Gender (female vs. male)	0.984 (0.611–1.586)	1.586		
Age (> 60 years vs. ≤ 60 years)	1.577 (0.972–2.557)	0.065		
Tumor location (right-sided colon vs. left-sided colon)	0.828 (0.498–1.377)	0.467		
Tumor size (> 5 cm vs. ≤ 5 cm)	0.987 (0.613–1.589)	0.957		
Tumor differentiation (poor vs. well to moderate)	1.550 (0.709–3.391)	0.272		
T category (T3-4 vs. T1-2)	1.942 (0.928–4.061)	0.078		
N category (N1-2 vs. N0)	2.524 (1.566–4.070)	< 0.001	2.633 (1.632–4.248)	< 0.001
Preoperative CEA (> 5 ng/mL vs. ≤ 5 ng/mL)^a^	1.726 (1.049–2.841)	0.032		
Preoperative CA199 (> 35 U/mL vs. ≤ 35 U/mL)^b^	1.486 (0.800–2.760)	0.210		
Adjuvant chemotherapy (yes vs. no)	1.607 (0.735–3.513)	0.235		
ER-β expression (high vs. low)^c^	1.163 (0.623–2.171)	0.636		

*HR* hazard ratio, *CI* confidence interval, *ER-β* estrogen receptor-β, *CEA* carcinoembryonic antigen

^a^The data of preoperative CEA level were available in 246 patients

^b^The data of preoperative CA199 level were available in 222 patients

^c^The IHC scores of ER-β expression were obtained in 257 patients

**Table 3:** Univariate and multivariate Cox regression analyses of prognostic factors for 5-year disease-free survival in patients with high and low ER-β expression

Variables	Low ER-β expression (*n* = 51)		High ER-β expression (*n* = 206)			
	Univariate HR (95% CI)	*P* value	Univariate HR (95% CI)	*P* value	Multivariate HR (95% CI)	*P* value
Gender (female vs. male)	1.054 (0.339–3.271)	0.928	0.999 (0.558–1.699)	0.998		
Age (> 60 years vs. ≤ 60 years)	1.313 (0.416–4.139)	0.642	1.604 (0.931–2.766)	0.089		
Tumor location (right colon vs. left colon)	1.282 (0.413–3.975)	0.667	0.767 (0.429–1.373)	0.373		
Tumor size (> 5 cm vs. ≤ 5 cm)	2.227 (0.616–8.419)	0.217	0.817 (0.476–1.402)	0.463		
Tumor differentiation (poor vs. well to moderate)	1.598 (0.206–12.423)	0.654	1.462 (0.626–3.415)	0.380		
T category (T3-4 vs. T1-2)	26.1 (0.030–22,868.187)	0.345	1.551 (0.733–3.283)	0.251		
N category (N1-2 vs. N0)	5.179 (1.633–16.418)	0.005	2.086 (1.224–3.556)	0.007	2.232 (1.309–3.808)	0.003
Nav1.5 expression (high vs. low)	1.830 (0.401–8.362)	0.435	4.155 (1.012–17.056)	0.048	4.653 (1.131–19.142)	0.033

*HR* hazard ratio, *CI* confidence interval, *ER-β* estrogen receptor-β

## Discussion

In the present study, we found that high Nav1.5 expression was associated with high preoperative CEA level and high ER-β expression in 269 patients with non-metastatic colon cancer. With a more than 10 years of follow-up, patients with high Nav1.5 expression, especially those with high ER-β expression, had significantly shorter DFS than patients with low Nav1.5 expression. Herein, we suggest that Nav1.5 expression may be served as a prognostic factor of postoperative DFS for patients with non-metastatic colon cancer.

Accumulating studies revealed that high Nav1.5 expression was detected in several types of carcinoma as compared with corresponding normal epithelial specimens by using IHC staining [13, 18, 26]. Our study also found that Nav1.5 was commonly expressed in colon cancer tissues with significantly higher IHC score than that of tumor-adjacent normal tissues (5.1 ± 3.5 vs. 3.5 ± 2.7, *P* < 0.001), which implied that Nav1.5 might be associated with tumor development. Besides, we observed that high Nav1.5 expression was associated with high ER-β expression. The biological effect of β-estradiol (E2) notably occurs by binding to estrogen receptor (ER). Recently, Hu et al. [27] established ER-knockout and wild-type mice models and demonstrated that E2 up-regulated Nav1.1, Nav1.8, and Nav1.9 mRNA expression depending on ER-β. Accordingly, abundant ER-β expression in cancer cells might partially account for a high level of Nav1.5 expression. However, little is known about the underlying molecular mechanisms nowadays. Bi et al. [28] hypothesized that estrogen-ER complex interacted with estrogen response element sequences located in the promoter region of sodium voltage-gated channel alpha subunit 9 (*SCN9A*) and exerted its regulatory potential. Experiments might be essential to elucidate the mechanism of E2-mediated regulation of Nav1.5 expression in cancer cells.

Compared with its carcinogenic role, the potential prognostic value of Nav1.5 was seldom highlighted previously. In breast cancer, a significant association was found between neonatal Nav1.5 expression and clinically assessed lymph node metastasis, which might associate with a poor prognosis [20]. Similarly, relative mRNA expression level of Nav1.5 in ovarian cancer with lymph node metastasis was obviously increased as compared with that in ovarian cancer without lymph node metastasis [13]. In the present study, high Nav1.5 expression represented as a prognostic indicator of low long-term survival rate for the patients with non-metastatic colon cancer. Nav1.5 expression had no significant association with DFS in the patients with low ER-β expression, whereas the survival outcomes of patients with Nav1.5 expression were significantly different in patients with high ER-β expression. As a transcriptional regulator through ER-α and ER-β, the extracellular application of E2 significantly increased the amplitude of Nav1.5 expression above the reference level in a dose-dependent manner in MDA-MB-231 breast cancer cells, leading to a decrease in cellular adhesiveness [29]. This suggested that the biological effects of E2 could be enhanced in colon cancer with high ER-β expression, where the carcinogenic functions of Nav1.5 were strengthened, and the prognostic values of Nav1.5 expression were subsequently highlighted in patients with high ER-β expression. Categorizing the colon cancer patients by combining ER-β expression status with Nav1.5 expression level would be useful for distinction of prognosis between groups.

Accumulating in vitro studies have illustrated that cancer invasive potential was both efficiently inhibited by local anesthetics or by genetically down-regulating Nav1.5 expression using small interfering RNAs (siRNAs), indicating that Nav1.5 might be served as an ideal anti-metastatic target in colon cancer [18, 30]. In addition, our present study showed that Nav1.5 expression was significantly lower in tumor-adjacent normal tissues than in tumor tissues. Although high expression was identified in 52.6% (41/78) paired tumor-adjacent normal tissues in the present study, previous study revealed that Nav1.5 expression was noticeably lower in adult liver and kidney [31]. Accordingly, the treatment targeting Nav1.5 could specifically suppress tumor invasiveness rather than severely damage the function of the liver and kidney [32, 33]. In the future, adequate animal experiments or clinical studies would be essential to determine the effect when administering Nav1.5 inhibitors in various dosages and delivery models.

Nevertheless, several limitations of this study should also be acknowledged. First, although the association of ER-β with Nav1.5 expression in colon cancer was preliminarily revealed, it seemed to be insufficient to reveal the comprehensive molecular interaction of Nav1.5 with ER-β accounting for the prognosis of colon cancer patients. Second, we did not discriminate the subtypes of Nav1.5 in our analysis, as the biological function between adult and neonatal Nav1.5 isoforms were relatively different [34, 35]. Especially, neonatal Nav1.5 was identified as a novel marker of the metastatic phenotype and a potential therapeutic target in human breast cancer [26]. Third, with a considerable long-term follow-up, the expression of Nav1.5 seemed to have no impact on OS of colon cancer patients in the present study. In fact, treatment strategies dealing with recurrent disease were always inconsistent; patients with recurrent lesions undergoing radical ablative treatment might achieve a longer OS than those without tumor resection, which meant postoperative recurrence no longer indicated death completely. Additionally, a subset of patients in the present study was confirmed to die due to other causes, including heart or aged diseases, which may have led to underestimating the impact of Nav1.5 expression on long-term OS. However, these confounding factors were difficult to be controlled in the retrospective study. Therefore, a large and multicenter prospective study might be required to substantiate the prognostic predicting value of Nav1.5 in colon cancer patients.

In conclusion, our data showed that Nav1.5 was highly expressed in colon cancer tumor tissues. The high expression of Nav1.5 was associated with high ER-β expression and was also identified as a predictor for low 5-year DFS rate in patients with non-metastatic colon cancer. These results may help clinicians develop adaptive treatment strategies for the colon cancer patients at high risk of recurrence.

## Abbreviations

ER-β: estrogen receptor-β
TMA: tissue microarray
IHC: immunohistochemistry
OR: odds ratio
HR: hazard ratio
CEA: carcinoembryonic antigen
VGSCs: voltage-gated sodium channels
E2: β-estradiol
DAB: 3,3′-diaminobenzidine tetrahydrochloride
CI: confidence interval
DFS: disease free survival
OS: overall survival
